# Pore Development during the Carbonization Process of Lignin Microparticles Investigated by Small Angle X-ray Scattering

**DOI:** 10.3390/molecules26072087

**Published:** 2021-04-06

**Authors:** Harald Rennhofer, Janea Köhnke, Jozef Keckes, Johannes Tintner, Christoph Unterweger, Thomas Zinn, Karl Deix, Helga Lichtenegger, Wolfgang Gindl-Altmutter

**Affiliations:** 1 Department of Materials Science and Process Engineering, BOKU-University of Natural Resources and Life Science, A-1190 Vienna, Austria; JaneaKoehnke@gmx.de (J.K.); johannes.tintner@boku.ac.at (J.T.); helga.lichtenegger@boku.ac.at (H.L.); wolfgang.gindl-altmutter@boku.ac.at (W.G.-A.); 2 Department of Materials Physics, Montanuniversität of Leoben, A-8700 Leoben, Austria; Jozef.Keckes@unileoben.ac.at; 3 Wood K plus–Kompetenzzentrum Holz GmbH, A-4040 Linz, Austria; c.unterweger@wood-kplus.at; 4 ESRF—The European Synchrotron, 38043 Grenoble, France; thomas.zinn@esrf.fr; 5 Institute of Material Technology, Building Physics and Building Ecology, TU Wien, A-1040 Vienna, Austria; karl.deix@tuwien.ac.at

**Keywords:** lignin, biobased carbon, porosity, X-ray scattering, SAXS

## Abstract

Application of low-cost carbon black from lignin highly depends on the materials properties, which might by determined by raw material and processing conditions. Four different technical lignins were subjected to thermostabilization followed by stepwise heat treatment up to a temperature of 2000 °C in order to obtain micro-sized carbon particles. The development of the pore structure, graphitization and inner surfaces were investigated by X-ray scattering complemented by scanning electron microscopy and FTIR spectroscopy. Lignosulfonate-based carbons exhibit a complex pore structure with nanopores and mesopores that evolve by heat treatment. Organosolv, kraft and soda lignin-based samples exhibit distinct pores growing steadily with heat treatment temperature. All carbons exhibit increasing pore size of about 0.5–2 nm and increasing inner surface, with a strong increase between 1200 °C and 1600 °C. The chemistry and bonding nature shifts from basic organic material towards pure graphite. The crystallite size was found to increase with the increasing degree of graphitization. Heat treatment of just 1600 °C might be sufficient for many applications, allowing to reduce production energy while maintaining materials properties.

## 1. Introduction

Technical lignin is one of the most abundant bioresources available for industrial application, with a global production of roughly 100 Mt/y [1]. It is a natural polymer featuring a wide variety of bonds and functional groups, although it is formed mainly from three precursors only, coumaryl, coniferyl and sinapyl alcohol [2]. Technical lignin, which arises as a by-product of the pulp and paper industry, was until recently used for fuel production mainly, but other applications for, e.g., binders or dispersants [3,4] and pharmaceutical and biomedical applications [5,6] are upcoming. Especially carbonization of lignin opens new possibilities for the production of, e.g., lignin-based carbon fibers [7], lignin-based carbon nanotubes [8] or carbon black [9,10], of which carbon black has clearly the largest market. Carbon black is derived on industrial scale from aromatic oils rich in hydrocarbons or from hydrocarbons directly [11], sometimes from other sources, like, e.g., carbides [12]. Carbon black has a wide field of applications, ranging from pigments and reinforcing filler materials for stability or enhanced electrical conductivity [13] to new energy applications like supercapacitors, batteries or even solar devices [11,12]. The applicability of carbon materials is highly dependent on material properties like electrical conductivity, porosity and pore size [12,14]. To enable applications of carbonized lignin as a substitute for carbon black from other sources, detailed knowledge of its properties is required. Lignin-based carbons are often characterized chemically or by spectroscopical means only [15], but little has been reported about morphology, including porosity [16,17]. The pore size in the nm range was, e.g., measured for activated carbons derived from lignin by N_2_ and CO_2_ sorption [18]. Anyhow, in most of the previous studies, small-angle X-ray scattering (SAXS) is often not considered, even though SAXS provides a powerful method for investigation of structure and morphology on the nanoscale. For instance, it was used to study pore volume fractions of micro- and mesopores of porous powders from CaCo_3_ [19]. It was also used in combination with small-angle neutron scattering to investigate pores in disordered porous carbon, where special care was taken of the evaluation procedure related to the porous nature of the graphitic material [20]. The necessity of SAXS to access buried porosity was shown, e.g., with the characterization of disordered carbons [21]. With the possibility of pore characterization, the method also features as an easily applicable tool to follow pore development, e.g., changing materials anisotropy and changing structural features in activated carbons from wood [22], the development of porosity in deposited charcoal [23], the charcoal properties dependent on pyrolysis temperature [24] or the change of aggregates in carbon black pastes with aging [25]. 

Recently, we reported on the production of micro-sized carbon black particles from different lignin sources, for which the applicability for electrically conductive polymer [26,27] or cellulose nanopaper [28] was investigated. In these publications, the focus was on the carbonization, the chemistry and the electrical properties of resulting polymers. In the present publication, we intend to follow up on the morphology with special respect to porosity of these lignin-based carbon particles. To this purpose, we applied SAXS together with wide-angle X-ray diffraction (WAXD) and complemented these techniques with scanning electron microscopy (SEM) and Fourier-transform infrared spectroscopy (FTIR). Some samples were additionally characterized by mercury porosimetry. The lignin samples were heat-treated at different elevated temperatures in order to carbonize and subsequently partially graphitize the material. The process is expected to change the materials morphology, especially the nanopores, but the question was, how the type of lignin influences the carbon black porosity at elevated temperatures and whether or not temperatures up to 2000 °C are required for highly porous materials.

## 2. Results

### 2.1. Scanning Electron Microscopy (SEM)

**Figure 1:**
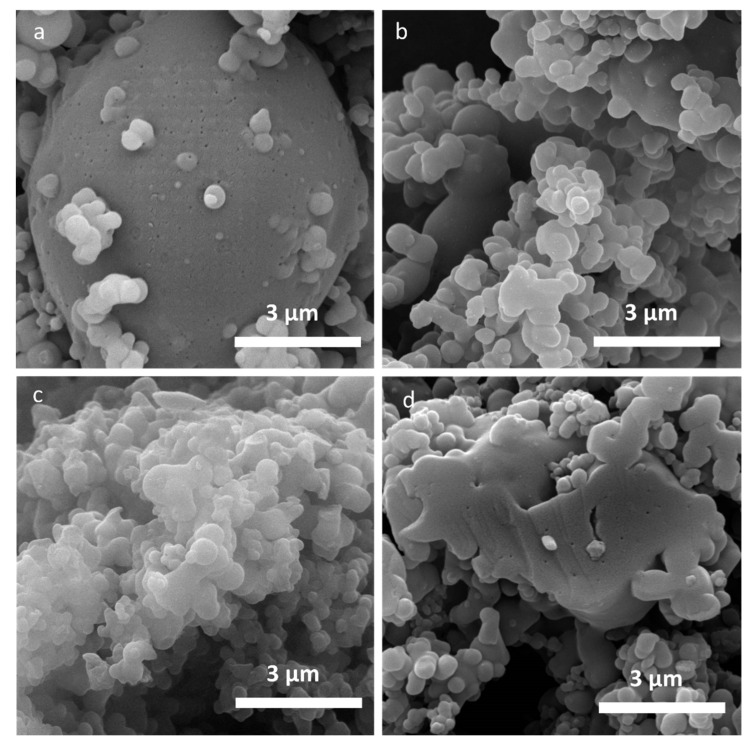
SEM micrographs of organosolv lignin samples, (**a**) O_250_; (**b**) O_800_; (**c**) O_1600_; (**d**) O_2000_.

**Figure 2:**
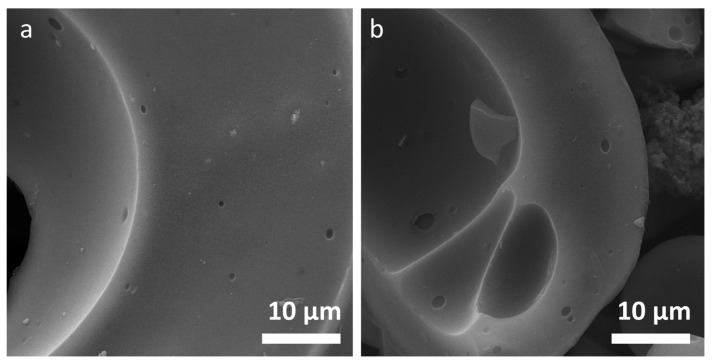
SEM micrographs of lignosulfonate samples, (**a**) L_250_; (**b**) L_2000_.

The images of organosolv lignin samples are depicted in Figure 1 for O_250_, O_800_, O_1600_ and O_2000_. Figure 1a shows micro-sized lignin particles with a certain size variation. The O_raw_ and O_250_ samples exhibit several submicron-sized pores that are visible in the surface of the bigger particles (Figure 1a). These openings, which are not found in the smaller particles surface (Figure 1a–c), are still visible after heat treatment at 2000 °C (Figure 1d). The sample O_2000_ still exhibits indication of open pores in the bigger structure, while these are not observed in the surface of the small spherical particles (Figure 1d). The overall shape of the organosolv particles stays spherical. Kraft and soda lignin-based samples exhibit similar features, i.e., open submicron pores (see Supplementary Information (Figure S1)). Lignosulfonate samples do not exhibit indication of open submicron pores, only few bigger openings in the µm range can be found. The morphology of surfaces in the µm range does not change with heat treatment. The samples feature smooth closed surfaces. As an example, two images of L_250_ and L_2000_ are depicted in Figure 2a,b, respectively. Organosolv and lignosulfonate images exhibit very different morphology in the submicron range. While organosolv features wider porous areas and small compact particles, lignosulfonate exhibits rather bulk-like bigger curved objects with some pores in the µm range.

### 2.2. Wide-Angle X-ray Diffraction (WAXD)

**Figure 3:**
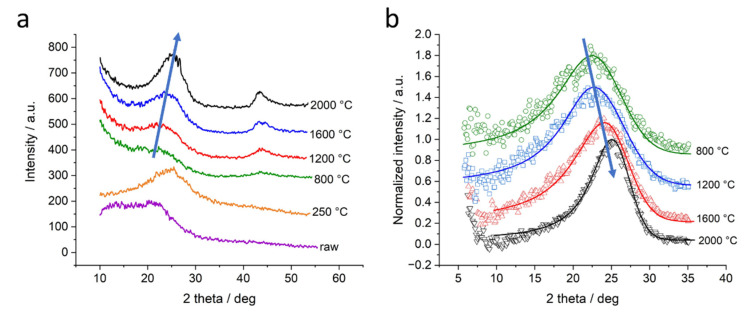
WAXD profiles of organosolv lignin-based samples. Curves are shifted by an arbitrary factor along the *Y*-axis: (**a**) curves of organosolv lignin-based samples for all temperatures; (**b**) fit function through (002)-peaks after subtraction of the low-angle signal of organosolv lignin-based samples displaying the shift and the narrowing of the (002)-peak with higher temperature. The peak shift is indicated with an arrow.

The WAXD curves of samples of all four lignins display very similar changes with increasing heat treatment temperature. As a representative example, the diffraction curves of organosolv lignin-based samples are displayed in Figure 3a. The diffraction curves of lignosulfonate, kraft and soda lignin-based samples can be found in the Supplementary Information (Figure S2). 

The raw lignin and the thermostabilized sample exhibit a broad intensity distribution that can be attributed to rather amorphous domains in the sample. There is no increase of the scattering curve towards low angles. For higher heat treatment temperatures, the diffraction curves change considerably. A peak structure emerges with peaks around scattering angles of 22.9° 2theta and 43.7° 2theta for the 800 °C sample. These peaks can be attributed to (002) and (100)/(101) of graphite, respectively. The (002)-peak becomes narrower and shifts towards higher scattering angles with higher heat treatment temperature. The (100)/(101)-peak becomes more pronounced but stays constant in its position with higher heat treatment temperature. For temperatures higher than applied during thermostabilization, the background at small diffraction angles also increases. This is due to the fact of the increasing small-angle X-ray scattering (SAXS) signal, i.e., the WAXD profiles are recorded on top of SAXS intensity [21]. In general, SAXS intensity at high scattering angles follows a power law [25,29], which is also found in porous carbons [21]. Thus, the background contribution was approximated by the following formula: I(2θ) ~ (2θ)^−n^ and subtracted before further data evaluation. The curves in Figure 3a display the WAXD data on top of the SAXS background, while the curves in Figure 3b are displayed after subtraction of the SAXS contribution. The shape and position of the (002)-peak was evaluated in detail with the following procedure. Since the peaks are not symmetric, a simple Gaussian fit could not be applied. Therefore, a combination of the Lorentzian and Gaussian functions was used for an asymmetric fit to determine peak position (2θ) and full width at half maximum (FWHM) of the (002)-peak. Details about the function can be found in the Supplementary Information. Figure 3b displays the fit curves of the organosolv-based samples without the SAXS background. The curves were shifted along the *Y*-axis to enhance visibility. It is visible that the right flank of the peaks towards higher diffraction angles only slightly changes with increasing temperature, while the peak position shifts and the left flank of the peaks changes, narrowing the peak. The peak position 2θ is related to the lattice spacing *d*_002_ of the graphite lattice and the wavelength *λ* via the Bragg equation:

(1) $$\lambda =2\cdot d_{002}\cdot sin\theta$$

The full width at half maximum (FWHM) of the peaks can be related to the size of coherently scattering domains *D*_002_, i.e., the lattice spread along the crystallographic axis indicated by the diffraction peak, by the Scherer equation, with k being a scaling factor, usually 0.9 for graphite (002) reflection [30], *λ*—the wavelength of X-rays, FWHM—in radians, *B*—correction for machine broadening and *θ*—from the position of the peak, then the size of coherently scattering domains *D*_002_ can be defined as follows:

(2) $$D_{002}=\frac{k\cdot \lambda }{\left(FWHM-B\right)\cdot cos\theta }$$

**Figure 4:**
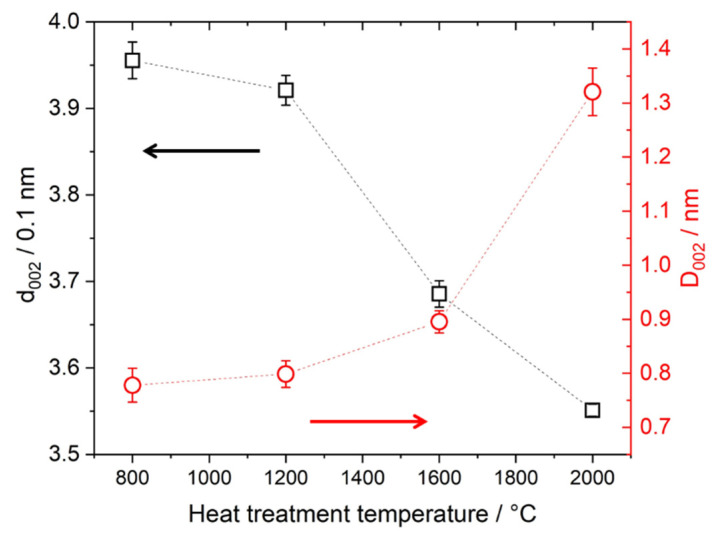
Result from the evaluation of wide-angle diffraction curves of organosolv lignin-based samples. The lattice spacing *d*_002_ (black squares, left axis) and the size of coherently scattering domains in the (002) direction *D*_002_ (red circles, right axis) are displayed. The connecting lines are for guiding the eye only, the arrows additionally indicate the corresponding axis. The errors indicated result from the fitting errors—the error of *d*_002_ at 2000 °C is within the displayed square and therefore not visible.

The values for *D*_002_ and *d*_002_ were calculated and results for organosolv lignin-based samples are depicted in Figure 4 and listed in Table 1. The value for *d*_002_ decreases from about 4.0 Å for the 800 °C sample to 3.6 Å for the 2000 °C sample, while *D*_002_ increases from about 0.8 nm to 1.3 nm.

The diffraction curves of the samples based on the three other lignins were evaluated accordingly. Data for lignosulfonate-based samples can be found in Table 1, the results of soda and kraft lignin-based samples can be found in the Supplementary Information (Table S1). The values of *D*_002_ and *d*_002_ are slightly different at the lowest heat treatment temperature (800 °C), ranging from *D*_002_ = 0.74 nm and *d*_002_ = 3.91 Å for K_800_ to *D*_002_ = 0.86 nm for L_800_ and *d*_002_ = 3.98 Å for S_800_. After heat treatment at 2000 °C, the *d*_002_ is quite comparable for all samples, with a value around 3.5 Å, while the apparent crystallite size *D*_002_ varies in the range of 1.21 nm to 1.45 nm. The average number of graphite layers *S* in the graphite stacking can be estimated by dividing the apparent crystallite size by the lattice distance [31]: *S = D*_002_/*d*_002_. The values of *S* can be found in Table 1 and Table S1. For the samples heat-treated at 800 °C, the values of *S* are quite comparable with *S* ≅ 2.0. After heat treatment at 2000 °C, the lignins exhibit comparable values of about 3.5–3.7, only S_2000_ exhibits a higher value of *S* = 4.2.

### 2.3. Small-Angle X-ray Scattering (SAXS)

**Figure 5:**
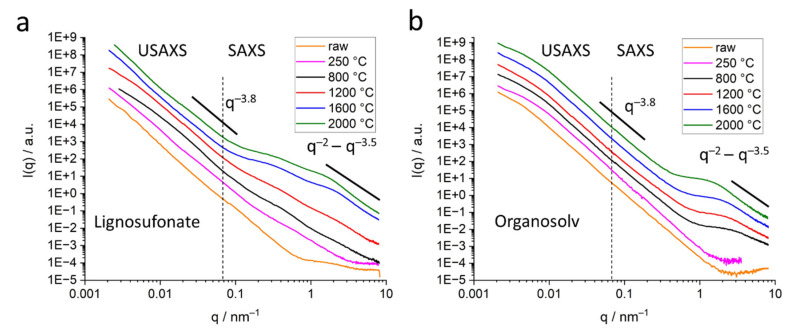
Small-angle X-ray scattering data of samples with different heat treatment—raw lignin, thermostabilized (250 °C), 800 °C, 1200 °C, 1600 °C and 2000 °C. The heat treatment temperatures are indicated in the graph. The curves are shifted along the *Y*-axis to enhance visibility; the curves displayed at higher intensity correspond to higher heat treatment temperature. The panels display scattering curves of (**a**) lignosulfonate; (**b**) organosolv. The q ranges of USAXS and SAXS are indicated by the dashed line. The slopes of the power law in the scattering curves are indicated.

SAXS data were recorded in the laboratory for all samples. The curves of organosolv, kraft and soda lignin-based samples were similar in display, i.e., only one pore population in the nm range was visible, exhibiting increasing pore diameter with increasing heat treatment temperature. Only the lignosulfonate-based sample exhibits a more complex scattering signal with pores of different sizes evolving with higher heat treatment temperatures. To get insight into the pore structure towards the length scale of several hundreds of nanometers, it was decided to perform ultra-small-angle X-ray scattering (USAXS) on a selection of samples. All samples from lignosulfonate and from organosolv were chosen for USAXS. Results of the scattering experiments over the full q range of USAXS and SAXS are depicted in Figure 5a (lignosulfonate) and Figure 5b (organosolv).

The lignosulfonate samples (Figure 5a) exhibit several shoulders along the scattering curves, indicating different populations of voids or pores. The raw lignin and the thermostabilized sample exhibit only slight shoulders. For higher temperatures, two distinct shoulders evolve at around 0.5 nm^−1^ and 2 nm^−1^, that become more pronounced with higher heat treatment temperature and shift slightly to lower *q*-values. In addition, in the q range below 0.1 nm^−1^, the shoulder signal is less pronounced. The organosolv samples (Figure 5b) exhibit a shoulder in the USAXS regime around 0.005 nm^−1^ for all samples, including for the thermostabilized lignin and, less pronounced, for the raw lignin. In the mid-q range (*q* = 0.03–0.2 nm^−1^), the curves follow a strict power law of *I*(*q*) ~ *q^−m^* with m about 3.8, indicating a smooth surface of the bigger voids. In the high-q-range (*q* > 1 nm^−1^), the samples heat-treated at higher temperatures exhibit a shoulder at about *q* = 1–2 nm^−1^ that can be attributed to pores/voids in the nm range. The shoulder shifts towards lower *q*-values with increasing heat treatment temperature, indicating an increase in pore diameter. The raw lignin and the thermostabilized samples do not exhibit an indication of such pores in the nm range.

Kraft and soda lignin-based samples exhibit indication of nm-sized pores similar to the organosolv samples. The respective curves can be found in the Supplementary Information (Figure S3a,b).

#### 2.3.1. Submicron Pores, the USAXS Regime

The USAXS modes of lignosulfonate and organosolv samples exhibit indication of pores in the sub-µm range (Figure 5a,b). For lignosulfonate samples, the shoulders at *q*-values below 0.1 nm^−1^ indicate a wide size distribution without a defined main pore size. The shoulders shape changes without exhibiting a clear trend. In contrast, organosolv samples exhibit a slight shoulder for O_raw_ and well-defined and similar features for O_thermo_ and all heat-treated samples around *q* = 0.008 nm^−1^. The size of these submicron pores was estimated by the Guinier approximation in a similar way as for the smaller pores—see below. The size was estimated with about 300 nm for O_raw_ and around 220 nm for all other organosolv samples. The shoulders of L_800_ and L_1200_ could be attributed to a wide distribution around the 150 nm pore size.

#### 2.3.2. Nanometer-Sized Pores, Classical SAXS Evaluation Strategy

For a more detailed evaluation of pores in the nm range the mid-q contribution (intensity in the range of *q* = 0.03–0.2 nm^−1^) following power law *I*(*q*) ~ *q^−m^* was determined by a fit extrapolated towards higher *q*-values and subtracted from the scattering data. The modified scattering intensities are indicated *I’*(*q*) *= I*(*q*) − *q^−m^* in the further text. The power law in the mid-q range is indicated in Figure 5, featuring exponents between 3.5 and 4, suggesting smooth surfaces of the bigger pores and interfaces. 

**Figure 6:**
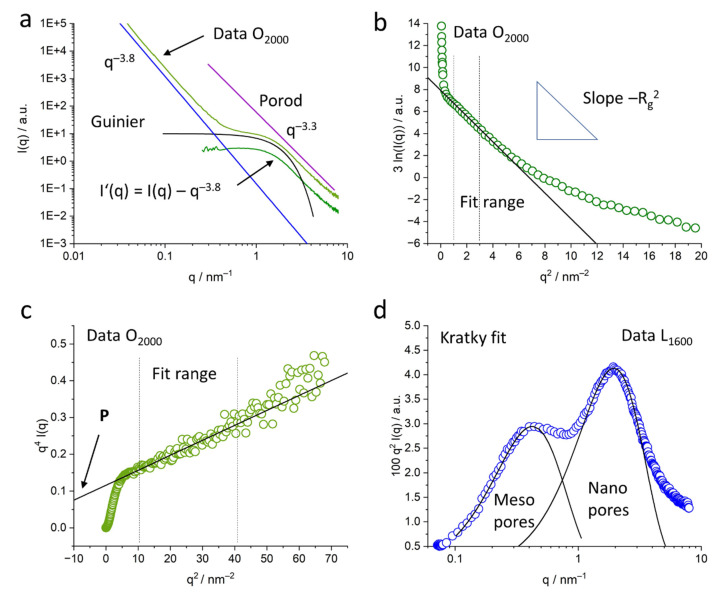
Display of the evaluation procedures applied to the data. (**a**) Original data *I*(*q*) of O_2000_ with the mid-q power law, Porod’s power law, modified scattering curve *I’*(*q*) and Guinier approximation of the pores; (**b**) Guinier fit to O_2000_ data to determine *R_g_* by a linear fit; (**c**) Porod fit to O_2000_ data in order to determine the Porod constant P from the intercept of the fit function with the *q*^4∙^*I*(*q*) axis; (**d**) Kratky plot for L_1600_ data with the Gaussian fit functions related to the meso- and nanopores.

Figure 6 displays an example for the data treatment and examples for the further applied steps in data evaluation. The corresponding formulas are described in the following text. Figure 6a displays the original scattering curve of O_2000_ with the mid-q power law of *q*^−3.8^ and the modified scattering curve after subtraction of this scattering contribution. It further displays the second power law of *q*^−3.3^ in the so-called Porod regime. A power law fit in this region of the scattering curve, the so-called Porod evaluation, is applied to access pore surface and surface roughness. Figure 6a also displays the so-called Guinier approximation for spherical pores to access the nanopore radius. Both methods are described below.

Guinier evaluation: The shoulders in the scattering curves *I’(q)* around 0.5–2 nm^−1^ can be related to nanopores and were evaluated with the Guinier approximation for spherical pores with radius *R* to get an indication of the size and evolution of pores [26]. If *I’(q)* is the modified scattering intensity, *A* is a constant and *R_g_* is the radius of gyration of the pores related to the actual pore radius *R* by *R*^2^ = 5/3 *R_g_*^2^, the following equation can be used:

(3) $$I^{\prime }\left(q\right)\;\sim \;A\cdot e^{-q^{2}R_{g}^{2}/3}$$

By taking the natural logarithm of the equation and multiplying it by 3, a linear equation of the form 3∙ln(*I*’) ~ −*R_g_*^2∙^*q*^2^ is achieved; that allows determining *R_g_* from the slope of a linear fit. The fit range was chosen in a strictly linear region of the data *q*^2^ = 1–3 nm^−2^. An example fit to the O_2000_ data together with the fit range indicated is displayed in Figure 6b.

Porod evaluation: In the high-q range above *q* = 3 nm^−1^, the scattering intensities *I*(*q*) and subsequently *I’*(*q*) follow, again, power law *I’*(*q*) = *P∙q^−n^* with *P* being the Porod constant proportional to the inner surface of the samples and n being the power exponent. The exponent *n* can be related to the surface roughness of the nanopores, with *n* being near 4 for smooth surfaces. Lower values indicate a rougher surface, with values towards 3 corresponding to a surface fractal and values between 2 and 3—to a mass fractal [29]. A power law fit was applied in the q range, from *q* = 3 to 6 nm^−1^ to determine n.

To evaluate the Porod constant *P*, an adapted Porod evaluation for power law exponents < 4 was chosen, where surface fluctuations with a contribution proportional to q^−2^ were taken into account [20,32]. If *P* is the Porod constant and *C* is the intensity factor related to surface fluctuations, the modified scattering curve can be described as follows:

(4) $$I^{\prime }\left(q\right)=P\cdot q^{-4}+C\cdot q^{-2}$$

By multiplication with *q*^4^, a linear equation of the form *q*^4∙^*I*’ = *C*∙*q*^2^ + *P* is achieved, that allows determining *P* by a linear fit in the data from the intercept of the fit function with the intensity axis. A linear fit in the range from *q* = 3 to 6 nm^−1^, i.e., with *q*^2^ ≅ 10 to 40 nm^−2^, was used in order to evaluate *P.* An example of the linear display of the data and the Porod fit is displayed in Figure 6c for the O_2000_ sample, indicating the fit range.

Kratky fit: From the scattering curves, the scattering invariant *Q* was calculated, which is a measure for the scattering of the sample material, related to the volumes of pores:

(5) $$Q=\;\int_{0}^{\infty }q^{2}\cdot I^{\prime }\left(q\right)\;dq$$

*Q* can be seen as the area below the curve in the so-called Kratky plot. These Kratky plots, *q*^2^ *I’*(*q*) as a function of *q*, emphasize the pore structure and allow further evaluation related to the nm pores only, since the modified scattering intensity *I’*(*q*) is used. An example of a Kratky plot is displayed in Figure 6d for the L_1600_ sample. Since the modified scattering data *I’*(*q*) were used for calculation of *Q,* the integration from zero to infinity can be determined by integration over the given data and extrapolation towards higher *q*-values with the Porod’s law using the exponent n. The intensity towards zero is negligible due to the previous subtraction of *q*^−m^.

For the lignosulfonate samples, the two contributions of the smaller nanopores and the somewhat bigger mesopores, both in the nm range were separated according to [19]. The areas corresponding to the individual peaks in the Kratky plot around 0.5 nm^−1^ and 2 nm^−1^ are related to the respective pore volumes *Q*_meso_ and *Q*_nano_ contributing to the scattering curves. The areas were determined by fitting two Gaussian functions to the data. An example of the fit functions is displayed in Figure 6d for the L_1600_ sample.

Specific inner surface equivalent: With *Q* and *P* determined, the quantity *P*/*Q* that is proportional to the specific inner surface *S*/*V* of the samples could be calculated. With *φ*_1_ being the volume fraction of pores with respect to the bulk material, *P*/*Q* is as follows:

(6) $$\frac{P}{Q}=\frac{1}{\pi \varphi_{1}\left(1-\varphi_{1}\right)}\cdot \frac{S}{V}$$

Since *φ*_1_ was not determined, *S*/*V* could not be calculated directly. We therefore present *P*/*Q* only.

#### 2.3.3. Kratky Plots

**Figure 7:**
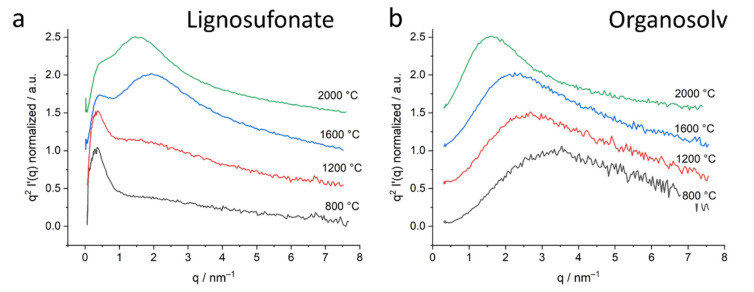
Kratky plots of the corrected small-angle X-ray scattering data of lignosulfonate (**a**) and organosolv (**b**) samples. Samples treated at 800 °C–2000 °C are displayed, the heat treatment temperatures are indicated in the graph. The curves are shifted along the *Y*-axis to enhance visibility.

The Kratky plots of lignosulfonate and organosolv samples are displayed in Figure 7a,b, for the kraft and soda samples in Figure S3c,d, respectively. 

It shall be noted that the plot in Figure 6d features a logarithmic q-axis, while the other plots have a linear scale. The logarithmic scale in Figure 6d was only chosen in order to display the fit functions of meso- and nanopores in a good resolution. Like the original scattering curve, the Kratky plots show two pore sizes in the nm range for the lignosulfonate samples and only one pore size for the organosolv samples at the given length scale. The linear q-scale clearly shows a considerably smaller contribution of mesopores of the lignosulfonate samples. While the peak at around 0.5 nm^−1^ related to mesopores does not change much with temperature, the peak at around 2 nm^−1^ increases with the increasing heat treatment temperature. The organosolv, kraft and soda samples exhibit only one peak, which evolves with heat treatment.

#### 2.3.4. Guinier Evaluation 

Spherical pores were chosen for the evaluation because of the remaining slope of the modified scattering curve towards small *q*-values. It is visible in Figure 6a and Figure 9a,b that the modified scattering curve exhibits a rather horizontal scattering profile towards low *q*-values in the log–log plot, i.e., the remaining power law of the sort *q*^−c^ with c << 1, which indicates spherical pores [29]. 

**Figure 8:**
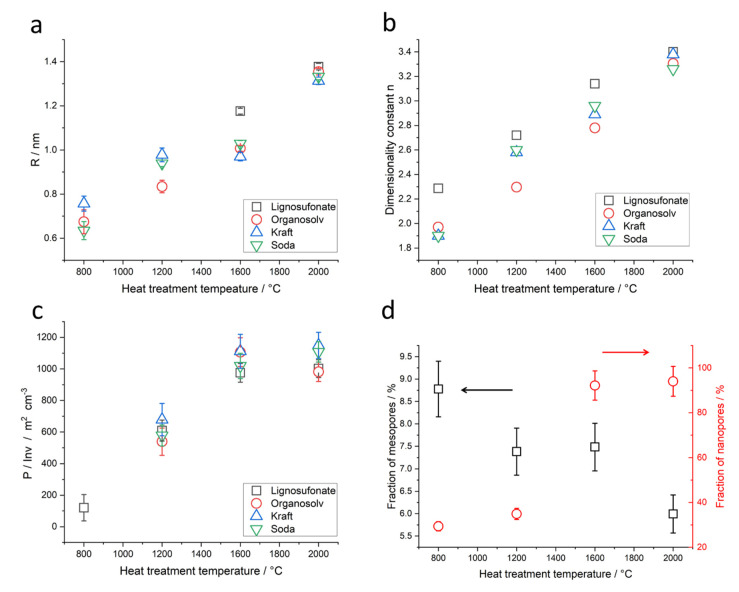
Results from the evaluation of small-angle X-ray scattering data: (**a**) radius of the nanopores; (**b**) dimensionality from Porod regime; (**c**) specific inner surface equivalent *P*/*Q*; (**d**) volume fraction related to mesopores (squares, left ordinate) and nanopores (circles, right ordinate).

The evaluation was only possible for samples with heat treatment of 800 °C or above. Results of samples derived from all four lignin sources are depicted in Figure 8a. For L_800_ and L_1200_, the Guinier evaluation could not be applied due to the not well-defined shoulders; these datapoints are thus missing in the plot. Organosolv, kraft and soda lignin-based samples at 800 °C feature pore radii of about 0.6–0.8 nm that increase with heat treatment temperature. While the rate of increase is different, the final pore size after heat treatment at 2000 °C is comparable for all samples, including lignosulfonate. The pore radius is about 1.35 nm and thus nearly doubles. The error indicated in the plot results from the fit and is below 2% of the displayed values for most of the samples.

#### 2.3.5. Specific Inner Surface: Porod Evaluation

It shall be noted that the Porod regime is not affected by the subtraction of the mid-q power law since the corresponding intensities are negligible for higher *q*-values. 

The values of the power law exponent n are depicted in Figure 8b for samples from all four lignin sources. K_800_ and S_800_ exhibit very small values with *n* ~ 1.9, the O_800_ exhibits a somewhat higher value, but still smaller than *n* = 2.0 and L_800_ exhibits a greater value of *n* = 2.3, indicating a different surface roughness of the nanopores. With higher heat treatment temperature, the samples exhibit greater values of n, i.e., smoother surfaces. Lignosulfonate exhibits a smaller increase with higher temperatures than the other lignin-based samples. At 2000 °C, all samples exhibit comparable values, *n* = 3.3 for O_2000_ and S_2000_ and somewhat higher *n* ~ 3.4 for K_2000_ and L_2000_. This indicates generally smoother surfaces of the nanopores. The error in n resulting from fitting is <2% of the given values, i.e., error bars would lay within the displayed symbols in Figure 8b and are thus omitted.

The results of the specific inner surface equivalent *P*/*Q* can be found in Figure 8c for all samples. Samples K_800_, S_800_ and O_800_ could not be evaluated by this method since the power law in the Porod regime is smaller than 2. The L_800_ sample exhibits a small value of *P*/*Q* with about 120 m^2^ cm^−3^ L_800_. At 1200 °C, the samples exhibit values around 600 m^2^ cm^−3^. The values increase at 1600 °C to about 1000–1100 m^2^ cm^−3^ and exhibit no further increase at 2000 °C. Errors indicated in the plot result from the fit. The heating step from 1200 °C to 1600 °C increases *P*/*Q* by a factor of nearly two for all samples, while the heating at 2000 °C does not change *P*/*Q* much anymore.

As mentioned above, the contributions of the smaller nanopores *Q*_nano_ and the somewhat bigger mesopores *Q*_meso_ were determined for the lignosulfonate samples. To better compare the evolution of the pore volumes, the contributions were related to the area sum at 2000 °C: the fraction of mesopores or nanopores for each temperature was thus calculated by *Q*_meso_/(*Q*_meso,2000_ + *Q*_nano,2000_) and also *Q*_nano_/(*Q*_meso,2000_ + *Q*_nano,2000_). The results are displayed in Figure 8d.

For L_800_, the fraction of the mesopore volume is about 9% and the fraction of the nanopore volume is about 30% of the volume measured at 2000 °C. L_1200_ does exhibit only slight changes, a decrease of mesopore volume fraction to 7.5% and nearly no increase in the nanopore volume fraction to about 35%. Heat treatment above 1200 °C clearly changes a lot in the pore volume. The mesopore fraction stays at 7.5%, while the nanopore fraction increases to about 90%. L_2000_ exhibits a slightly increased nanopore volume fraction of about 94% while the mesopore volume fraction decreases to 6%.

#### 2.3.6. Nanopore Size Evaluation in Real Space: Radial Distribution Function

Since the size of nanopores and mesopores could not be evaluated properly for all the lignosulfonate samples, a second approach besides the Guinier approximation was chosen. A fit with the GNOM program [33] of the ATSAS package [34] was applied to the corrected data in the nanopore range in order to evaluate the changes in pore size due to heat treatment. With GNOM, the size distribution function *p*(*r*) = 4π/3∙*r*^3^∙*N*(*r*) for polydisperse spheres with radius *r* and relative number of spheres with this radius *N*(*r*) was determined, where *p*(*r*) relates to the corrected scattering intensity *I’*(*q*) as follows [35]:

(7) $$I^{\prime }\left(q\right)=4\pi \int_{0}^{D}\gamma \left(r\right)\frac{\sin\left(qr\right)}{qr}r^{2}dr$$

and *p*(*r*) is related to the correlation function *γ*(*r*) of the particles as follows: *p*(*r*) = *r*^2^ *γ*(*r*). 

**Figure 9:**
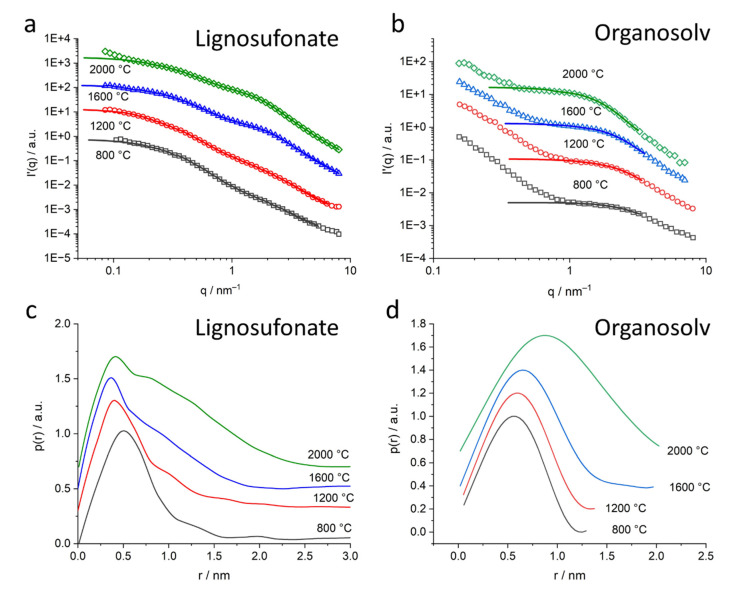
Evaluation of the size distribution function *p*(*r*) for the nanopores of lignosulfonate and organosolv samples. The scattering contribution of bigger structures has been fitted by a power law and subtracted to access the nanopores previous to the evaluation. (**a**) Lignosulfonate scattering data (symbols) together with the fit result (solid line); (**b**) organosolv scattering data (symbols) together with the fit result (solid line); (**c**) corresponding *p*(*r*) results of lignosulfonate; corresponding *p*(*r*) results of organosolv (**d**).

The corresponding modified scattering curves together with the fit curves representation in *I’*(*q*) are displayed in Figure 9a,b for lignosulfonate and organosolv samples, respectively. The curves *p*(*r*) are displayed in Figure 9c,d for lignosulfonate and organosolv samples, respectively. Kratky and soda-based samples were not considered for the pair distribution analysis since the pore structure is simple and thus sufficiently described by the Guinier approximation.

The intensities *I’*(*q*) still exhibit some contribution in the low-q range not attributed to the nanopores that was not subtracted by the power law modification due to the complex nature of the scattering signal in the mid-q range. In the GNOM fit, this was not taken into account. The two wide shoulders of lignosulfonate samples (Figure 9a) and the one shoulder of the organosolv samples (Figure 9b) are approximated well by the fit curves. The *p*(*r*) curves of lignosulfonate (Figure 9c) show the main peak around the 0.5 nm pore radius and a wide size distribution towards higher pore radii that increases with heat treatment temperature. L_800_ exhibits rather a main distribution up to 1-nm size, whereas L_1200_ exhibits a shoulder up to 1.5 nm that is more pronounced for L_1600_ and L_2000_. Pore size of this main contribution increases up to 2.5 nm. Figure 9d shows the *p*(*r*) functions of organosolv samples, featuring a well-defined peak shifting from 0.5 nm for O_800_ to 1.2 nm for O_2000_. The mean pore size of the nanopores increases and the size distribution widens. There were no considerable contributions of well-defined bigger pores found by the fit.

The complex pore size distribution of lignosulfonate-based samples was, in addition, addressed by mercury porosimetry. The resulting pore size distributions can be found in the Supplementary Information (Figure S6). The pronounced peaks can be found in all samples, namely, in the pore diameters of 27.5 µm for L_250_ and L_1200_ and 17 µm for L_1600_ and L_2000_. These big pores can be observed in the SEM pictures as well. The ranges of diameters can be specified from 5 µm to 100 µm for all samples. Pores smaller than 5 µm were only measured in very small proportions.

### 2.4. Fourier-Transform Infrared Spectroscopy (FTIR) 

**Figure 10:**
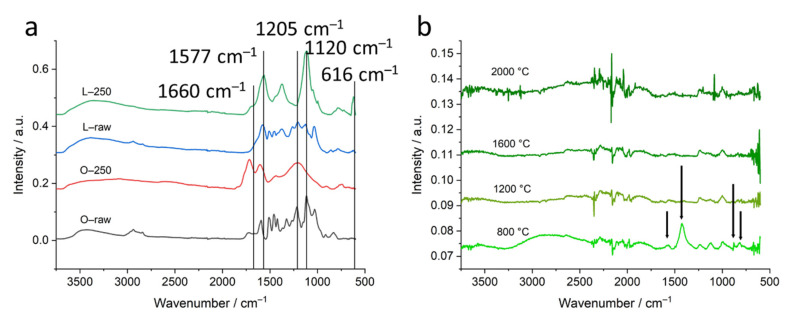
FTIR curves measured for lignosulfonate and organosolv samples heat-treated at different temperatures. Panel (**a**) displays curves of samples L_raw_ and L_250_ together with curves of samples O_raw_ and O_250_. Panel (**b**) displays curves of lignosulfonate samples heat-treated at higher temperatures. Lines and arrows indicate peaks discussed in the text.

FTIR measurements exhibit quite similar features for organosolv, kraft and soda raw lignins, while raw lignin from lignosulfonate exhibits considerably different features. This also holds true for thermostabilized samples. For temperatures higher than 800 °C, the main features of the FITR spectra disappear and spectra of samples based on different lignins look quite similar. Therefore, only spectra of samples of raw lignin and thermostabilized lignin from organosolv and lignosulfonate were chosen together with the higher-temperature spectra of organosolv-based carbons for display in Figure 10. A comparison of the FTIR spectra of all four raw and thermostabilized samples can be found in the Supplementary Information (Figure S4a,b). The FTIR spectra of the lignosulfonate lignin for all temperatures is displayed in Figure S4c,d. The FTIR spectra of kraft and soda lignin-based samples for the high-temperature treatment can be found in the Supplementary Information (Figure S5a,b). 

The main features of the FTIR spectrum of the organosolv lignin were reported previously [26]. For the changes with thermal treatment, we found, in addition, the following: the different raw lignins revealed specific differences of the C=O stretch vibration around 1660 cm^−1^. Two notable peaks in the lignosulfonate lignin arose at 1577 and 1205 cm^−1^. Both peaks are between other lignin peaks common in all four raw materials. They were observed by [36] in the lignosulfonate lignin, but were not assigned separately. It can be assumed that they are caused by aromatic skeletal vibration and aromatic ring breathing [37,38]. Among the thermostabilized spectra, again, lignosulfonate displays exceptional bands that can be assigned to the SO stretch (1120 cm^−1^) and the SO bend (616 cm^−1^) [39]. The pyrolysis temperature of 800 °C leads to a remarkable spectrum for lignosulfonate. In comparison to the other lignin types, the spectrum displays additional bands at the positions 1428 cm^−1^ and 878 cm^−1^ (long arrows in Figure 10b). Both band positions can be assigned to calcite. Further band maxima are found around 1568, 1223, 1119, 1003 and 820 cm^−1^. Positions 1568 and 820 cm^−1^ (short arrows in Figure 10b) as well as 878 cm^−1^ can be assigned to aromatic ring stretching and aromatic CH out-of-plane deformation vibration [40]. These aromatic bands disappear at even higher temperatures reducing FTIR spectra to minimized signals. This corresponds to the common transformation pattern of organic matter into graphite-like structures.

## 3. Discussion

The four different lignins were subjected to thermostabilization and different heat treatment temperatures up to 2000 °C. In WAXD, we observe similar behavior for all four lignins: after thermostabilization, the diffraction curves exhibit a broad peak related to the (002) reflection of graphite. The broad peak indicates a suboptimally ordered structure and possibly comparable small coherently scattering units. With temperatures above 800 °C, the material becomes more and more ordered, peaks shift towards higher scattering angles and narrow down (Figure 3). As an example, the organosolv peaks shift in position, from 21.3° to 24.9°, which corresponds to a d_002_ shifting from 4.17 Å to 3.58 Å, i.e., a decreasing lattice constant. In addition, the FWHM is reduced from 13.9° to 6.3°, which corresponds to an increase in lattice size D_002_ in the related direction, i.e., from 0.54 nm to 1.22 nm. By dividing *D*_002_ by *d*_002_, one can estimate the mean number of graphite layers in crystalline ordered stacks, which would thus increase from 1.3 to 3.4 layers. The other lignins exhibit similar changes with slightly different parameters; while *d*_002_ is smaller, the lattice spread is bigger. In general, one observes larger crystalline domains in the (002) direction featuring smaller lattice distance induced by heat treatment (Figure 4). This effect is documented for many carbon-based materials, from carbon fibers [41] to graphitic nanosheets [42] and graphene-like materials [31]. For lignin-based carbon fibers, a similar decrease in *d*_002_ was observed [7]: *d*_002_ was reported to be about 3.9 Å after heat treatment at 900 °C and decreased to about 3.5 Å after the 2000 °C treatment. Two effects are likely to account for the changes in the WAXD pattern, an increase in the layer lattice size and a reduction of amorphous and stacking fault contributions. It is suggested by [43] that an increasing in-plane lattice size in graphite accordingly changes the energetically favorable lattice spacing *d*_002_. Indeed, we observed an emerging (100)/(101)-peak which suggested a spread and organization of the lattice in this direction. While this effect was reported for pure graphite, the less ordered system of thermostabilized lignin (*d*_002_ > 4 Å) will also yield amorphous contributions and less ordered graphite-like arrangements. Thus, the poorly aligned material might dominate the scattering signal at lower temperatures and would thus be mainly responsible for the changes visible. Figure 3b shows, e.g., that the change in peak shape is mainly due to the fact of reduced intensity towards smaller scattering angles, while the flank of the Gaussian-shaped peak is nearly constant towards high angles at about 25–27°. This indicates that rather larger lattice distances in not well-ordered carbon arrangements are changed towards a better order, while the already ordered structures stay nearly constant. For sure, the emerging (100)/(101)-peak indicates the more graphitic nature of samples treated with temperatures towards 2000 °C. As shown previously [28], the samples with higher heat treatment temperature show higher electrical conductivity. Thus, the increased electrical conductivity can be related to the more graphitic nature of the material.

The SAXS signal clearly shows the difference in the nanostructure of the different lignin samples. While organosolv, kraft and soda-based samples exhibit a simple pore size distribution, lignosulfonate-based samples feature various pores on different length scales, the diameter of which changes considerably for higher heat treatment temperature. Figure 5b shows the scattering curves of the organosolv samples: the pores with about 200–300 nm size can also be observed for raw lignin and the thermostabilized sample, while the nanometre-sized pores are only visible for samples heat-treated at temperatures higher than 800 °C. This is also visible in the low-angle contribution of the WAXD curves (Figure 3a) that feature no additional background from SAXS contributions for the raw and thermostabilized curves. After subtraction of the low-q small-angle signal, the change in the nm-sized pores could be directly addressed: spherical pores increase in the mean diameter from about 0.7 nm to about 1.5 nm. Pores in the 1–2 nm range have also been reported in activated carbons of co-solutions activated by H_3_PO_4_ and heat treatment at 400 °C [18]. An increase of the nm-sized pores from lignin precursor to carbonized material after heat treatment at 800 °C was reported by [10] for kraft lignin. A wider pore size distribution ranging from 18 to 40 nm and higher was reported for iron oxide particles coated with carbonized lignin. Here, carbonization was carried out at 500 °C, but residual lignin was dissolved afterwards, which might have influenced the surface pore characteristics. An increase in pore size is generally observed with higher temperatures for carbon materials [12]. The specific inner surface equivalent of the nanopores increases from about 600 m^2^ cm^−3^ at 1200 °C to 1100 m^2^ cm^−3^. The dimensionality parameter evaluated in the Porod regime increases as well, which is interpreted as a change in surface roughness, i.e., the surface of the nanopores becomes smoother, from a mass fractal at temperatures below 1600 °C towards a surface fractal for samples treated with 1600 °C or 2000 °C [29]. This can be understood by the change in the carbon material towards a more graphitic structure. Better arrangement of previously unordered carbon material opens pores and better order of the crystallites results in smoother surfaces of the pores. A change in the stacking is also the basis of the changing contribution of surface fluctuations *q*^−2^, which was chosen as basis for the Porod evaluation [32]. Between the moderate-temperature treatments (800 °C, 1200 °C) and higher-temperature treatments (1600 °C, 2000 °C), the samples exhibit a considerable change in most of the structural parameters, i.e., *d*_002_ and *D*_002_ (Figure 4, Table 1, Table S1), *R* (Figure 8a), most prominently visible in *P*/*Q* (Figure 8c) and also the nanopore fraction of lignosulfonate (Figure 8d). This can be attributed rather to a physical change in the samples than a chemical one, since the FTIR data (Figure 10, Figures S4 and S5) suggest that the main change in chemistry occurs between 800 °C and 1200 °C. The general FTIR pattern of thermostabilized spectra corresponds well to the pyrolysis degree revealed between 250 and 300 °C [24]. The formation of calcite in lignosulfonate (two bands at 1428 cm^−1^ and 878 cm^−1^) at pyrolysis temperature of 800 °C can be explained as CO_2_ from the pyrolysis decomposition of organic matter trapped in nanopores reacts with CaO as a residue of CaSO_4_ as an intermediate product into CaCO_3_ [24,44]. Above the 1000 °C pyrolysis temperature, the removal of all elements except carbon is asymptotically achieved [24].

While the shape of the pores is generally not important for a generalized Guinier evaluation, the subtraction of the mid-q contributions allowed a more specific data interpretation. The modified scattering curve *I’*(*q*) shows a power law close to *q*^−0^, which indicates spherical pores. This is in contrast to porous carbons from, e.g., carbides, that exhibit slit-like pores [12], which results from the different basic material. The fit to the data of the lignin-based carbons in this work is in good agreement with the assumption of spherical pores (Figure 6a and Figure 9). It is interesting to see the influence different lignin materials have on the pore size and pore size distribution even after full carbonization at 1600 °C and 2000 °C. This is visible not only in the USAXS data in the low-q range (Figure 5), but also in the *p*(*r*) curves evaluated by the GNOM fit (Figure 9). Lignosulfonate displays bigger pore structures, evolving in a non-linear way with heat treatment, with a wider pore size distribution in the nm range. The main pore size of nm-sized pores stays nearly constant around 0.5 nm, while more and bigger pores towards 8 nm evolve. This wide and complex pore distribution also did not allow a valid Guinier evaluation for all lignosulfonate samples, thus the evaluation with GNOM was applied. In contrast, the organosolv-based samples show a nearly constant size of the submicron pores of about 220 nm and a clear trend in the development of the nm-sized pores towards bigger pores. Only the 2000 °C sample exhibits a considerable increase in the width of the *p*(*r*) distribution. Kraft and soda samples also exhibit a similar trend in the scattering curves, suggesting a pore development comparable to organosolv samples.

The observations from mercury porosimetry (Figure S6) complement the measurements from SAXS (Figure 5 and Figure S3): nm-sized pores that increase in amount and diameter and a complex pore structure towards length scales in the micrometer regime are shown by SAXS. The mercury porosity results show the main pore size in the μm range. Mercury porosimetry displays a negligible number of pores in the 10–100-nm range and the measurement signal close to the resolution of the equipment. Thus, the two methods give full insight into the pore structure only when used together. The results also show good agreement with the SEM images, where the 10–20 μm pores are visible as the hollow spheres, typically for the lignosulfonate samples. The smaller pores found by mercury porosimetry are the holes in these structures. 

In our previous work, it was shown that the electrical conductivity of the samples increases with heat treatment temperature [28]. While kraft, soda and organosolv lignins exhibit an increase by a factor of about 10 (50–500 Ω^−1^ mm^−1^) in electrical conductivity, among the samples heat-treated at 800 °C and 2000 °C, the lignosulfonate lignin does exhibit a comparatively smaller maximum value of about 50 Ω^−1^ mm^−1^ at 2000 °C. This might not only be due to the different microscopic arrangement (lignosulfonate and kraft samples exhibit larger spherical particles), but could also be influenced by the much higher porosity and bigger pores in the material. It was also observed that electrical conductivity increases most between 800 °C and 1600 °C, while no considerable increase was observed between 1600 °C and 2000 °C. This behavior corresponded to the development of crystal structure and pores. Such observations could be crucial for tailoring the lignin-based carbon black properties with the lowest amount of processing energy necessary.

## 4. Materials and Methods

### 4.1. Materials

Four different technical lignins were used as raw material: kraft lignin (Indulin AT) from MeadWestvaco^®^, soda lignin (Protobind 2400^®^) purchased from GreenValue^®^, softwood lignosulfonate bought from Borregaard^®^ and organosolv lignin provided by Fraunhofer^®^.

### 4.2. Lignin Carbonisation

Lignin was thermostabilized at 250 °C in a Memmert oven with a heating rate of 0.01 °C min^−1^ and ambient atmosphere. Thermostabilization causes oxidative changes in the lignin structure and results in thermoset lignin. Carbonization was performed in a GERO HTK8 oven equipped with a 61 graphite retort at an argon gas flow rate of 150 L/h. Four different target temperatures were accomplished for all lignins: 800 °C, 1200 °C, 1600 °C and 2000 °C. The heating rate was 1 °C min^−1^ up to 500 °C with a 1-h holding step at 500 °C. Afterwards, the heating rate was increased to 5 °C min^−1^ with 1-h holding steps at 900 °C and the final temperature. For better understanding, we refer to the samples with a letter and the temperature subscribed throughout the text. Thus, organosolv raw, thermostabilized, and heat-treated afterwards with either 800 °C, 1200 °C, 1600 °C or 2000 °C samples are denoted as O_raw_, O_250_, O_800_, O_1200_, O_1600_ and O_2000_, respectively. Lignosulfonate samples are denoted as L_raw_, L_250_, L_800_, L_1200_, L_1600_ and L_2000_, soda—as S_raw_, S_250_, S_800_, S_1200_, S_1600_ and S_2000_ and kraft—as K_raw_, K_t250_, K_800_, K_1200_, K_1600_ and K_2000_, accordingly.

### 4.3. Characterization of Samples

Scanning electron microscopy (SEM) was conducted using a QuantaTM 250 field-emission environmental scanning electron microscope equipped with a Schottky field emission gun which operated at 10 kV in high vacuum of 1 × 10^−6^ mbar. The samples were sputter-coated with gold and images were taken at different magnifications with the secondary electron detector (ETD).

Wide-angle X-ray diffraction (WAXD) was carried out with a 5-circle X-ray diffractometer equipped with Cu-Kα source with a parabolic multilayer mirror in the primary beam and a secondary graphite monochromator and a wavelength of *λ* = 0.15418 nm. Intensity data were documented in a step of 0.02° between 2θ = 5° and 50° with 4 s dwell time.

Small-angle X-ray scattering (SAXS) was performed with a Rigaku S-Max3000 3-pinhole SAXS camera with an MM002+ source (Cu-Kα, *λ* = 1.54 Å) and a Triton 200 multiwire detector. Scattering images were recorded in the q range 0.08–8 nm^−1^, with q being the magnitude of the scattering vector related to the scattering angle 2θ and the wavelength *λ* by the Bragg equation: *q* = 4π/*λ* sin *θ*. All 2D data were azimuthally integrated to obtain 1D scattering profiles, i.e., scattering intensity vs. *q*, *I*(*q*) and later background subtracted for further analysis [26]. For select samples, additional ultra-small-angle X-ray scattering (USAXS) measurements were performed on ID02 beamline at the ESRF, Grenoble, France. Scattering images were taken for a 10-m and a 31-m sample-to-detector distance with a maximum exposure time of 0.01 s using a Rayonix MX 170 HS detector. The settings result in a *q* range from 0.002 to 0.765 nm^−1^. The background-subtracted synchrotron data could be combined with the SAXS data from the laboratory allowing for a full *q* range of 0.002–8 nm^−1^.

Attenuated total reflection Fourier-transform infrared spectroscopy (ATR-FTIR) was performed with 4 cm^−1^ resolution on a Helios spectrometer (Ultrafast Systems BV, Amsterdam, the Netherlands) equipped with a diamond crystal. The spectra were baseline-corrected before further evaluation. 

Mercury porosimetry was used on a set of lignosulfonate-based samples. To cover a large pore radius range, two devices, namely, Pascal 140 and Pascal 440 (both Porotec), were used. The former generates pressures up to 400 kPa and covers pores down to 5 µm, the second—down to 5 nm due to the high pressure of up to 400 MPa. Measurements were performed with the pressure increase speed of 6–19 MPa/min at 21 °C

## 5. Conclusions

In this work, porous carbons from four different lignin sources, organosolv, lignosulfonate, kraft and soda, were investigated. The atomic arrangement and morphology on the nm scale were studied dependent on different heat treatment temperatures up to 2000 °C. Heat treatment results in a graphite-like structure, that becomes more graphitic with higher temperatures, i.e., the lattice distance d_002_ decreases and the lattice spread in this crystal direction increases. This is attributed to a growth of the basic scattering units, both in-plane and in the (002) direction. The rearrangement of the carbon atoms and graphene sheets leads to an increase in the pore size of the nm-scaled pores and a wider pore size distribution. The nm-sized pores also exhibit smoother surfaces with higher heat treatment temperatures. Most investigated parameters exhibit the strongest development between 1200 °C and 1600 °C suggesting that heat treatment at 2000 °C might not be necessary under all circumstances. The knowledge about nm porosity and its development with temperature is highly important for applications of carbon materials in, e.g., the energy sector. The full characterization of cheap lignin-based carbons with this respect will thus foster the applicability in future.

## Figures and Tables

**Table 1:** Apparent crystallite size *D*_002_ and lattice spacing *d*_002_ evaluated from the FWHM and the peak position 2θ of the (002)-peak, respectively. The corresponding heat treatment temperature (HTT) and the resulting number of graphite layers *S* is indicated. The errors from the fit are <0.5% for the peak position and <4% for the FWHM, resulting in an error estimate of maximum ± 0.02 in *d*_002_, of maximum ± 0.04 in *D*_002_ and of about ± 0.1 in *S*.

	Organosolv					Lignosulfonate				
HTT	2θ	FWHM	*d*_002_	*D*_002_	*S*	2θ	FWHM	*d*_002_	*D*_002_	*S*
°C	°	°	Å	nm		°	°	Å	nm	
800	22.5	10.0	3.96	0.78	2.0	22.8	9.1	3.90	0.86	2.2
1200	22.7	9.7	3.92	0.80	2.0	23.2	8.8	3.84	0.88	2.3
1600	24.2	8.7	3.69	0.90	2.4	24.6	8.7	3.62	0.89	2.5
2000	25.1	6.0	3.55	1.32	3.7	25.4	6.5	3.50	1.21	3.5

## Data Availability

Data are contained within the article or Supplementary materials.

## Supplementary Materials

The following are available online, Figure S1: SEM images of kraft and soda lignin samples; Figure S2: Additional WAXD curves; Figure S3: Additional SAXS data; Figure S4: Additional FTIR data 1; Figure S5: Additional FTIR data 2; Figure S6: Pore distribution of lignosulfonate samples measured by mercury porosimetry; Table S1: WAXD data of kraft and soda lignin samples.

## Sample Availability

Sample material is still partly available from the authors.
