# Supplementary material for: Pore Development during the Carbonization Process of Lignin Microparticles Investigated by Small Angle X-ray Scattering
Source: Molecules. 2021 Apr 6;26(7):2087. doi: 10.3390/molecules26072087 (PMC8038752; doi:10.3390/molecules26072087)
Supplement: Supplementary file 1 [file molecules-26-02087-s001.pdf]

Supplemental Information

Pore development during the carbonization process of lignin microparticles investigated by Small angle X-ray scattering

Harald Rennhofer*, Janea Köhnke, Jozef Keckes, Johannes Tintner, Christoph Unterweger, Thomas Zinn, Karl Deix, Helga Lichtenegger, Wolfgang Gindl Altmutter

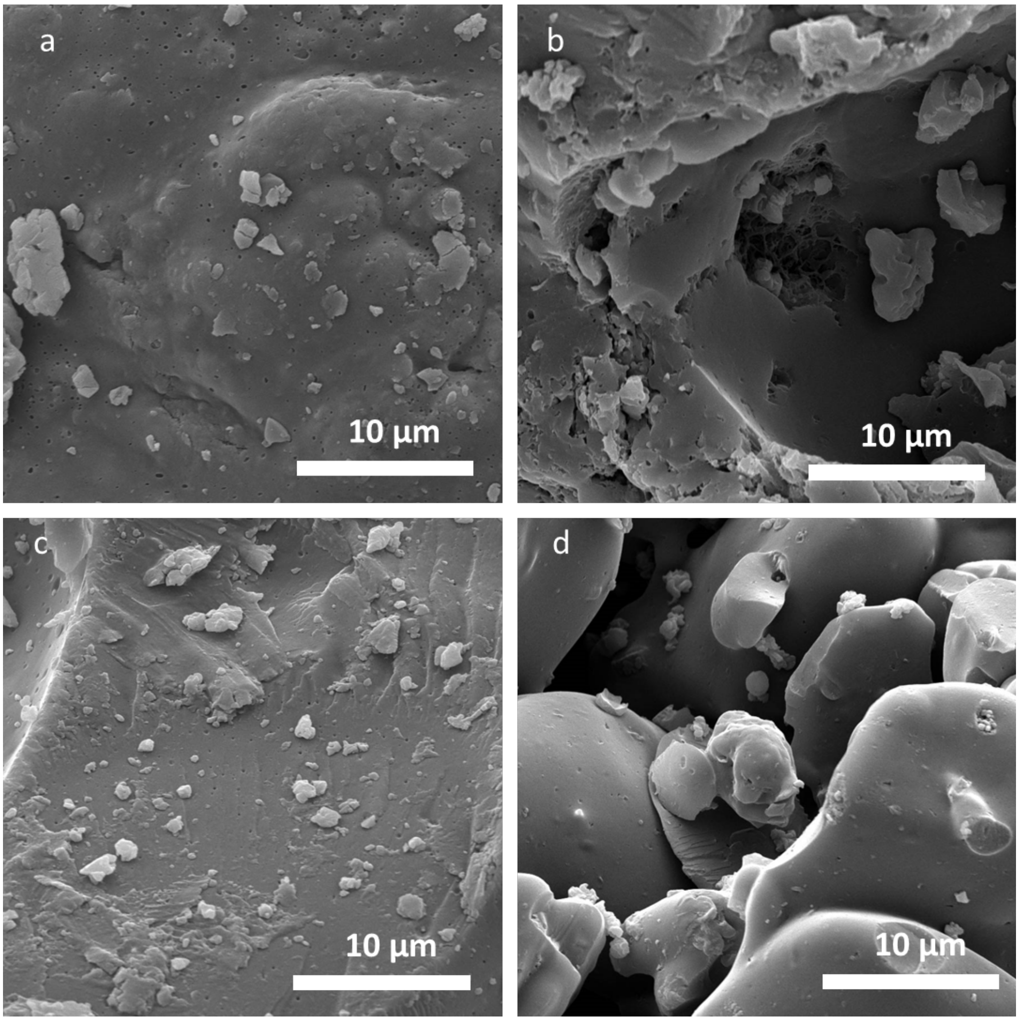

Figure S1: SEM images of Kraft and Soda lignin samples: (a) K_{raw}; (b) K_{2000}; (c) S_{raw}; (d) S_{2000}. The open sub-micron pores are visible for all samples.

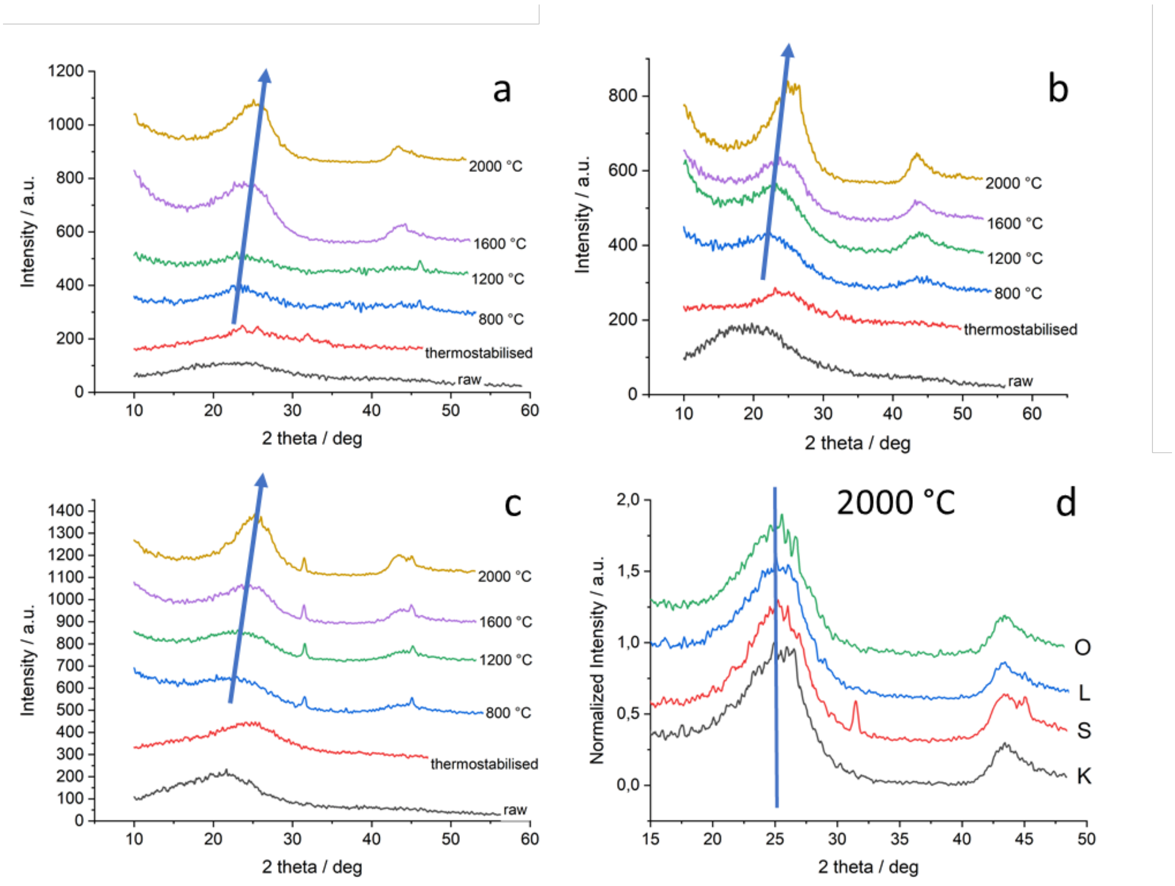

Figure S2: Additional WAXD curves. (a) Lignosulfonate; (b) Kraft lignin; (c) Soda lignin; (d) samples O_{2000}, L_{2000}, S_{2000} and K_{2000} in comparison. All cures show a shift of the (002) peak towards higher diffraction angles, indicated by the arrow and a narrowing of the (002) peak, with higher heat treatment temperature. The peaks in panel (d) display the same position (indicated by the line) and same shape of the (002) peak.

An asymmetric function A(x) was constructed from a simple Lorentzian function L(x) and a simple Gaussian function G(x), with the constraint of same amplitude at the peak position x_0:

$$A(x) = \begin{cases} L(x) \;\; for \;\; x < x_0 \\ G(x) \; for \;\; x > x_0 \end{cases}$$

With the two functions:

$$L(x) = \frac{A}{(x - x_0)^2 + w^2}$$

And

$$G(x) = \frac{A}{w^2} \, e^{-\frac{(x-x_0)^2}{s^2}}$$

With *A* the scaling factor and the parameters *w* and *s* controlling the widths of the two functions. Then the full width at half maximum *FWHM* can be determined analytically by the simple relation:

$$FWHM = w + s\sqrt{-\ln(0.5)}$$

Table S1: WAXD data of kraft and soda lignin samples: The heat treatment temperature (HTT), peak position (2θ), peak width in degrees (FWHM), lattice spacing (d_{002}), apparent crystallite size (D_{002}) and stacking parameter (S) are given in the table. Errors from the fit are < 0.5 % for the peak position and < 4 % for the FWHM, resulting in an error estimate of maximally +/- 0.02 in d002, of maximally +/- 0.04 in D002 and of about +/- 0.1 in S.

	Kraft					Soda				
HTT / °C	2θ / °	FWHM / °	d_{002} / Å	D_{002} / nm	S	2θ / °	FWHM / °	d_{002} / Å	D_{002} / nm	S
800	22.7	10.4	3.91	0.74	1.9	22.3	10.1	3.98	0.77	1.9
1200	22.8	8.9	3.90	0.88	2.2	23.0	9.4	3.88	0.83	2.1
1600	24.1	8.6	3.69	0.91	2.5	24.5	8.0	3.64	0.97	2.7
2000	25.5	6.2	3.50	1.29	3.7	25.6	5.5	3.48	1.45	4.2

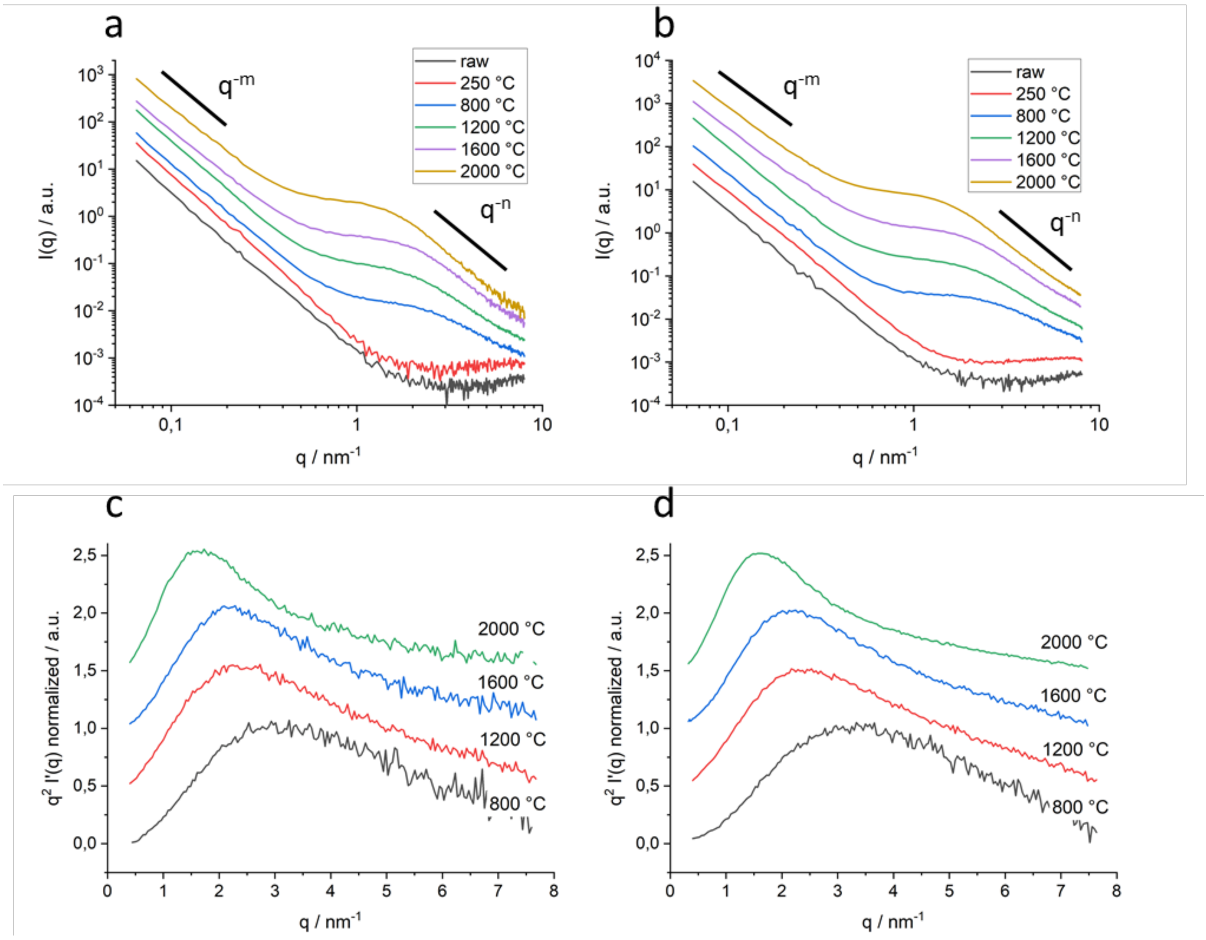

Figure S3: Additional SAXS data. Small angle scattering curves of (a) kraft scattering curves; (b) soda scattering curves; (c) Kratky plot of kraft samples; (d) Kratky plot of soda samples.

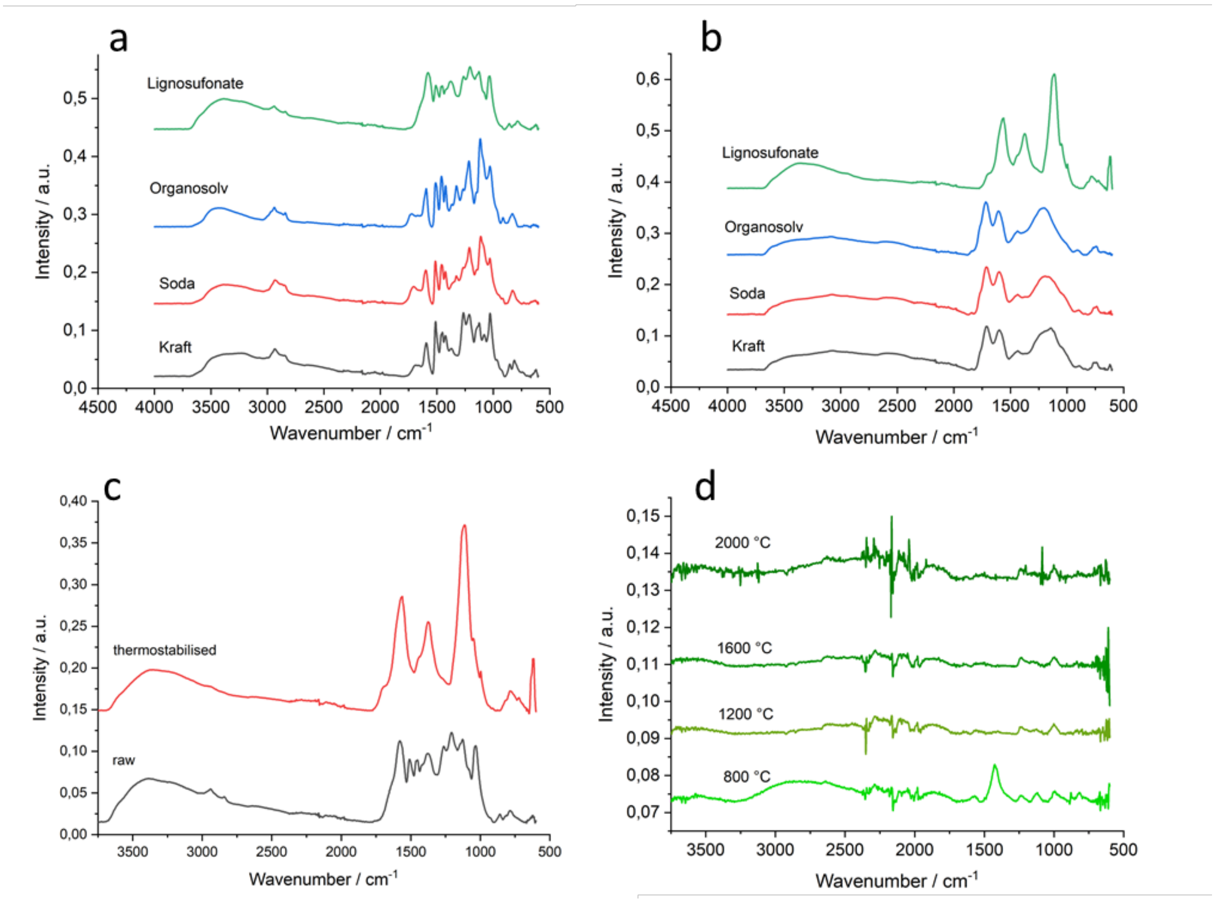

Figure S4: Additional FTIR data 1: (a) FTIR spectra of the raw lignins; (b) spectra of the thermostabilized samples; (c) low temperature lignosulfonate spectra; (d) high temperature lignosulfonate samples.

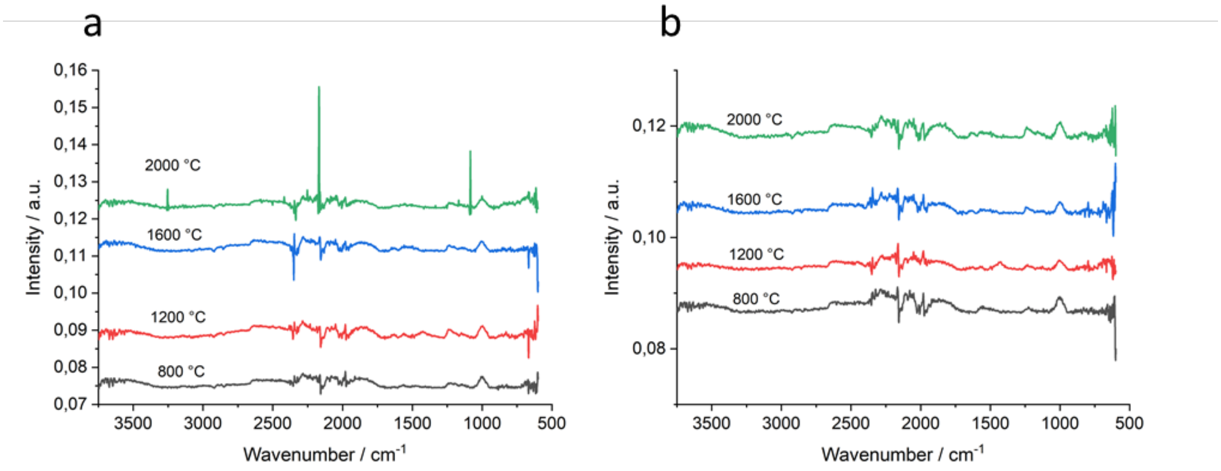

Figure S5: Additional FTIR data 2: (a) FTIR spectra of heat treated kraft samples; (b) FTIR spectra of the heat treated soda samples.

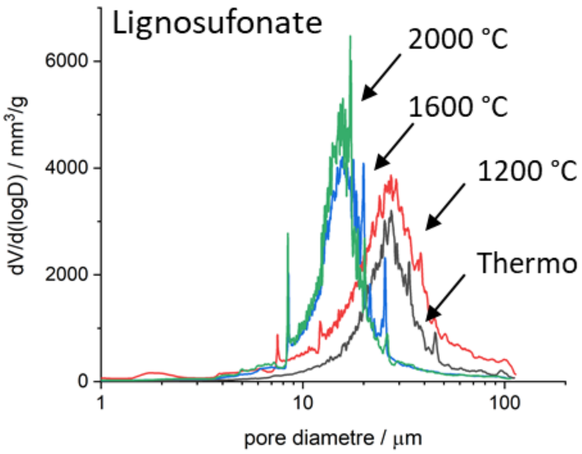

Figure S6: Pore distribution of lignosulfonate samples measured by mercury porosimetry. Changes in pore size distribution are visible with changing temperature. Values smaller than 1 μm could in principal be measured but are not relevant with respect to noise and resolution of the equipment.
